# Validation of single nucleotide polymorphisms potentially related to R-CHOP resistance in diffuse large B-cell lymphoma patients

**DOI:** 10.20517/cdr.2024.10

**Published:** 2024-05-24

**Authors:** Gabriele Perrone, Luigi Rigacci, Giandomenico Roviello, Ida Landini, Alberto Fabbri, Lorenzo Iovino, Benedetta Puccini, Emanuele Cencini, Enrico Orciuolo, Monica Bocchia, Alberto Bosi, Enrico Mini, Stefania Nobili

**Affiliations:** ^1^Department of Health Sciences, University of Florence, Florence 50139, Italy.; ^2^Research Unit of Hematology, Department of Medicine and Surgery, Campus Biomedico University, Rome 00128, Italy.; ^3^Unit of Hematology, Azienda Ospedaliera Universitaria Senese, University of Siena, Siena 53100, Italy.; ^4^Unit of Hematology, Santa Chiara University Hospital, University of Pisa, Pisa 56126, Italy.; ^5^Clinical Research Division, Fred Hutchinson Cancer Center, Seattle, WA 98109-4433, USA.; ^6^Unit of Hematology, Careggi University-Hospital, Florence 50134, Italy.; ^7^Department of Experimental and Clinical Medicine, University of Florence, Florence 50134, Italy.; ^8^Department of Neuroscience, Psychology, Drug Research and Child Health, University of Florence, Florence 50139, Italy.; ^#^Authors contributed equally.

**Keywords:** Diffuse large B-cell lymphoma (DLBCL), R-CHOP regimen, host genetics, single nucleotide polymorphism (SNP), biomarkers, tumor drug resistance

## Abstract

**Aim:** Diffuse large B-cell lymphoma (DLBCL) is the most common B-cell non-Hodgkin lymphoma (NHL). Despite the availability of clinical and molecular algorithms applied for the prediction of prognosis, in up to 30%-40% of patients, intrinsic or acquired drug resistance occurs. Constitutional genetics may help to predict R-CHOP resistance. This study aimed to validate previously identified single nucleotide polymorphisms (SNPs) in the literature as potential predictors of R-CHOP resistance in DLBCL patients, SNPs.

**Methods:** Twenty SNPs, involved in R-CHOP pharmacokinetics/pharmacodynamics or other pathobiological processes, were investigated in 185 stage I-IV DLBCL patients included in a multi-institution pharmacogenetic study to validate their previously identified correlations with resistance to R-CHOP.

**Results:** Correlations between rs2010963 (*VEGFA* gene) and sex (*P* = 0.046), and rs1625895 (*TP53* gene) and stage (*P* = 0.003) were shown. After multivariate analyses, a concordant effect (i.e., increased risk of disease progression and death) was observed for rs1883112 (*NCF4* gene) and rs1800871 (*IL10* gene). When patients were grouped according to the revised International Prognostic Index (R-IPI), both these SNPs further discriminated progression-free survival (PFS) and overall survival (OS) of the R-IPI-1-2 subgroup. Overall, patients harboring the rare allele showed shorter PFS and OS compared with wild-type patients.

**Conclusions:** Two out of the 20 study SNPs were validated. Thus, these results support the role of previously identified rs1883112 and rs1800871 in predicting DLBCL resistance to R-CHOP and highlight their ability to further discriminate the prognosis of R-IPI-1-2 patients. These data point to the need to also focus on host genetics for a more comprehensive assessment of DLBCL patient outcomes in future prospective trials.

## INTRODUCTION

Diffuse large B-cell lymphoma (DLBCL) is the most common B-cell non-Hodgkin lymphoma (NHL), accounting for about 30% of all NHL cases^[1]^. Two classifications, i.e., the 5th edition of the WHO Classification of the hematolymphoid tumors (WHOHAEM5)^[2]^ and the International Consensus Classification (ICC)^[3]^, are today available, both holding the classification of DLBCL based on the cell of origin (COO) that was initially obtained by gene expression profiling (GEP)^[4]^. COO subtypes have been then further stratified by providing additional subtypes with specific clinical features^[5-7]^. Today, surrogates of GEP, i.e., immunohistochemical (IHC) algorithms, are used to evaluate the COO, thus discriminating between germinal center B-cell (GCB) subtype, characterized by a better prognosis, and activated B-cell (ABC) (i.e., non-GCB) subtype (e.g., the Hans algorithm^[8]^). Other tumor biomarkers are today commonly evaluated, including CD20, Ki-67, as well as MYC, BCL2, and BCL6, since a worst prognosis is predicted by the co-occurrence of MYC rearrangements with BCL2 and/or BCL6 rearrangements (i.e., “double-hit” and “triple-hit” lymphoma, respectively)^[9,10]^.

From the clinical point of view, the DLBCL prognosis is established by the International Prognostic Index (IPI), available in several versions, that includes clinical and pathological characteristics^[10-12]^. Following the introduction of rituximab in the CHOP (i.e., doxorubicin, vincristine, cyclophosphamide, and prednisone) regimen, the revised IPI (R-IPI) has been proposed^[13]^.

However, despite these relevant molecular differences among DLBCL subtypes as well as the variability in terms of clinical and pathological features, the mainstay of the pharmacological first-line treatment is represented by the chemo-immunotherapeutic regimen R-CHOP in which the anti-CD20 monoclonal antibody rituximab is added to the CHOP regimen. R-CHOP has been approved based on its higher efficacy in comparison with the CHOP regimen in several clinical trials performed about 20 years ago^[14-16]^, the results of which have been successively confirmed^[17,18]^. R-CHOP is routinely administered to stage I-IV DLBCL patients. However, about 30%-40% of patients show intrinsic or acquired resistance to R-CHOP. Through the years, efforts have been made to overcome the occurrence of R-CHOP resistance. R-CHOP dose intensification^[19,20]^ and the addition/replacement of drugs in R-CHOP have been investigated. R-CHOP dose intensification failed to show an advantage over standard R-CHOP either when patients underwent 8 cycles instead of 6 standard cycles^[19]^ or when 14-day versus the standard 21-day cycles were compared^[20]^. The main examples of R-CHOP modified regimens are Pola-R-CHP (i.e., polatuzumab vedotin-piiq, rituximab, cyclophosphamide, doxorubicin, prednisone)^[21]^ and the dose-adjusted EPOCH-R (i.e., etoposide, prednisone, vincristine, cyclophosphamide, doxorubicin plus rituximab)^[22,23]^. The Pola-R-CHP regimen showed an advantage in terms of progression-free survival (PFS) compared with R-CHOP^[21]^. However, both these treatments are offered only to high-risk double- or triple-hit lymphoma DLBCL and are characterized by more severe side effects compared with R-CHOP^[21,22]^. Thus, the percentage of patients who are candidates for a pharmacological treatment other than R-CHOP is low since double- or triple-hit lymphomas occur in less than 10% of cases of DLBCL^[24]^.

Overall, the currently available clinical and molecular prognostic algorithms should be integrated with other determinants for an earlier identification of patients at risk of developing disease progression during or at the end of up-front treatment (intrinsic drug resistance) or at risk of relapsing after initial response to R-CHOP (acquired drug resistance)^[25]^.

A less explored field in this context, yet one that could contribute to this aim, concerns the germline genetics of DLBCL patients. Through the years, some pharmacogenetic clinical trials have investigated a relevant number of single nucleotide polymorphisms (SNPs) in genes involved in pharmacokinetics/pharmacodynamics of drugs included in R-CHOP as well as in host immunity or inflammation processes, as determinants potentially predictive of response to this regimen. Results of these studies suggested SNPs in several genes (e.g., *ABCB1*, *ABCC2*, *ABCG2*, *SLC2A1*, *GSTP1*, *NCF4*, *IL10*, *VEGFA*)^[26-31]^ potentially related to the clinical efficacy of R-CHOP.

Interestingly, most of the products of the genes whose polymorphisms have been studied in the host counterpart and that have been linked to the outcome of DLBCL patients treated with R-CHOP in such pharmacogenetic studies, play a well-established role in the tumor counterpart of the drug resistance phenomenon^[32]^. For instance, doxorubicin, vincristine, and cyclophosphamide are substrates of ATP Binding Cassette transporters (i.e., Pg-P, MRP2, and BCRP, which are encoded by *ABCB1*, *ABCC2*, and *ABCG2*, respectively), whose overexpression leads to the extrusion of these drugs from tumor cells, resulting in the occurrence of intrinsic or acquired multidrug resistance^[33,34]^. On the other hand, transporters involved in the cellular uptake of drugs (e.g., doxorubicin), such as membrane solute carrier (SLC) transporters, have been shown to be downregulated in cancer cells, including in DLBCL^[35]^. Even high expression levels of detoxicant enzymes [e.g., glutathione S-transferases (GST)] have been related to tumor drug resistance, including to doxorubicin, vincristine, and cyclophosphamide^[36]^. In addition, resistance to rituximab has been linked to several genes involved in the major pathways of its action, i.e., complement-dependent cytotoxicity (CDC), antibody-dependent cellular cytotoxicity (ADCC), and apoptosis induction (e.g., BCL2)^[37]^.

The presence of SNPs in the above-mentioned genes has been frequently associated with increased or decreased levels of their mRNA and/or protein expression levels, which ultimately reduce the response to drug treatment^[32]^.

Germline variants in genes known to contribute to tumor drug resistance due to their involvement in pharmacokinetics or pharmacodynamics of anticancer drugs have been investigated in several tumors besides lymphomas^[38-40]^. In particular, correlations have been identified between SNPs and resistance to various cytotoxic agents, such as anthracyclines and/or alkylants in breast cancer^[41-43]^, osteosarcoma^[44]^, and multiple myeloma^[45]^; fluoropyrimidines, irinotecan, or oxaliplatin in colorectal cancer^[46,47]^; platinum compounds in non-small cell lung cancer (NSCLC)^[48]^, esophageal cancer^[49]^, urothelial carcinoma^[50]^, and osteosarcoma^[44]^. Further associations have been pointed out between SNPs and resistance to methotrexate in childhood acute lymphoblastic leukemia^[51]^ or to gemcitabine in relapsed or refractory lymphoid malignancies^[52]^. Additionally, SNPs have been found to be associated with resistance to targeted agents (e.g., gefitinib in NSCLC^[53]^, sorafenib and regorafenib in hepatocellular carcinoma^[40]^, and imatinib in gastrointestinal stromal tumors^[54]^).

Findings on DLBCL pharmacogenetics have been recently reviewed by us^[55]^ and other authors^[56]^. However, most of the available studies lack a validation phase, hindering the potential contribution of these biomarkers in predicting drug response. One significant obstacle may stem from the difficulties in recruiting an independent cohort of DLBCL patients who have received uniform R-CHOP treatment, along with available clinical and pathological information.

Thus, the aim of this study was to validate the role of previously identified SNPs in predicting resistance to R-CHOP, in an independent suitable case series of DLBCL treated with R-CHOP that we enrolled in the framework of a pharmacogenetic study^[57]^.

## METHODS

### Patients

This study was performed on a cohort of 185 newly diagnosed stage I-IV DLBCL patients, uniformly treated with R-CHOP, who were prospectively enrolled in a previously published multi-institution pharmacogenetic GWAS study^[57]^. The protocol of the mentioned published pharmacogenetic study^[57]^ was approved by the local IRB (Careggi University Hospital, Florence, Italy) of the coordinator center (Prot. 2012/0033535) and by those of the participant centers. All patients provided written informed consent. The current study was performed by using data collected in the previous pharmacogenetic study^[57]^. The genotypes of patients for the study SNPs were obtained by the array we uploaded on the NCBI GEO repository as GSE186441^[57]^. Inclusion and exclusion criteria are reported by Perrone *et al.*^[57]^. According to the R-IPI^[15]^, patients were classified into three prognostic groups: R-IPI 0 (very good prognosis), R-IPI 1-2 (good prognosis), and R-IPI 3-5 (poor prognosis), as described by Perrone *et al.*^[57]^.

### Selection of SNPs

Based on literature data, 20 SNPs in 16 genes, previously identified as potential predictors of R-CHOP response, were investigated to validate such correlations. Study SNPs were selected according to the following criteria: (i) PubMed search of the following terms: “diffuse large B-cell lymphoma/DLBCL”, “polymorphisms”, “rituximab”, only English language publications, no time limits; (ii) only papers showing statistically significant correlations (*P* < 0.05) between SNPs and R-CHOP outcome; (iii) only papers including more than 65 patients; (iv) only SNPs included in the GSE186441 array. The PubMed search obtained 88 items, i.e., 8 reviews; 19 papers out of scope; 9 papers investigating tumor tissue; 3 papers performed with CHOP only; 3 papers performed with less than 65 patients; 30 papers with various issues (e.g., polymorphisms other than SNPs, no statistically significant associations between SNPs and R-CHOP efficacy, no #rs indication and/or not available full publication). Overall, 16 papers satisfied the planned criteria. In addition, available reviews were analyzed to identify papers and SNPs that would have been missed by the above-mentioned search or SNPs whose potential role in pharmacokinetics/pharmacodynamics of drugs included in R-CHOP is widely recognized. The above search identified 28 SNPs. Overall, 8 of them were not present in the array. The 20 SNPs considered for this validation analysis were present in genes codifying ATP-binding cassette transporters (i.e., *ABCB1* rs10276036^[58]^; *ABCB1* rs1128503^[58,59]^; *ABCC2* rs17222723^[27]^; *ABCG2* rs2231142^[60]^; *ABCG2* rs2231137^[29]^) and other transporters (*SLC2A1* rs841853^[31]^; *SLC2A1* rs1385129^[31]^), in genes codifying metabolism/detoxification enzymes (*GSTP1* rs1695^[61]^; *CBR1* rs20572^[62]^), in genes related to oxidative stress (*NCF4* rs1883112^[29]^), *CYBA* rs4673^[27]^; *RAC2* rs13058338^[29]^), in genes playing a role in hypoxia (*HIF1A* rs11549467^[31]^), in DNA repair (*APEX1* rs1130409^[31]^), in apoptosis (*LTA* rs1041981^[63]^, *TP53* rs1625895^[28]^), in immunity (*IL10* rs1800871^[26,64]^), and in angiogenesis genes (*VEGFR2* rs1870377^[65]^; *VEGFA* rs3025039^[30]^; *VEGFA* rs2010963^[30]^). By convention^[66]^, the most prevalent allele has been considered wild-type.

### Statistical analysis

Correlations between clinical and pathological characteristics or objective response (established by Cheson standardized response criteria as reported in^[57]^) and SNPs were evaluated by the Chi-Square test. The effect of SNPs on PFS or overall survival (OS) was evaluated by hazard ratios (HR) and corresponding 95% confidence intervals (CI), estimated by COX proportional hazard model. HRs were adjusted for gender and R-IPI. Dominant, recessive, and additive genetic models were considered; the best-fitting model was selected according to Wald Χ2-test. A *P* value < 0.05 (two-sided) was adopted as the significance threshold. To lower the chance of false positive discoveries, only concordant effects (same effect, same genetic model) were considered significant. To further evaluate the potential contribution of the candidate SNPs in refining the prediction of prognosis, a stratified analysis by R-IPI was also performed. Survival analysis was calculated by Kaplan-Meier method, and log-rank (Mantel-Cox) test was used to test the differences between genotypes. The statistical analysis was performed by the SPSS software v.28 and SNPassoc package (RStudio v. 2023.06.0). *P* value < 0.05 was considered statistically significant.

## RESULTS

### Patient characteristics

Overall, 185 DLBCL Caucasian patients were included in the analysis. As reported by Perrone *et al.*^[57]^, the median age was 59.17 years (range 22-83). Patients were well balanced for sex (50.3% male). Percentages of patients according to stages (stage I-II 45.4%; stage III-IV 54.6%) and R-IPI (R-IPI very good 14.1%; R-IPI good 56.8%; R-IPI poor 29.2%) were representative of this setting of disease. Performance status [Eastern Cooperative Oncology Group (ECOG)] was 0 in 62.2%, 1 in 31.4%, and 2 in 6.5%. Median PFS and OS had not been reached at the time of analysis. Median follow-up was 45 months (range 2.9-79.2).

The minor allele frequency (MAF) of the study cohort as well as for Caucasians and Asian subjects are reported in Table 1. Overall, the MAF observed in the study cohort was highly comparable with the Caucasian MAF. Some differences were observed, as expected, with the Asian MAF.

**Table 1:** Minor allele frequencies of the 20 study SNPs in the DLBCL patient cohort, in European and Asian populations

**Genes and functions**	**SNP**	**MAF**		
		**Study (*n* = 185)**	**European**	**Asian**
ATP-binding cassette transporters				
*ABCB1*	rs10276036	0.478	0.428	0.374
*ABCB1*	rs1128503	0.469	0.427	0.373
*ABCC2*	rs17222723	0.118	0.061	0.004
*ABCG2*	rs2231142	0.051	0.103	0.296
*ABCG2*	rs2231137	0.0	0.057	0.316
Glucose transporter				
*SLC2A1*	rs1385129	0.175	0.212	0.321
*SLC2A1*	rs841853	0.388	0.311	0.246
Metabolism/detoxification enzymes				
*CBR1*	rs20572	0.067	0.115	0.233
*GSTP1*	rs1695	0.318	0.326	0.192
DNA repair enzyme				
*APEX1*	rs1130409	0.435	0.468	0.404
Regulator of the adaptive response to hypoxia				
*HIF1A*	rs11549467	0.002	0.008	0.028
Immunity molecule				
*IL10*	rs1800871	0.32	0.238	0.317
Apoptosis-related genes				
*LTA*	rs1041981	0.310	0.327	0.451
*TP53*	rs1625895	0.197	0.139	0.029
Oxidative stress-related genes				
*CYBA*	rs4673	0.378	0.347	0.086
*NCF4*	rs1883112	0.345	0.448	0.48
*RAC2*	rs13058338	0.244	0.141	0.00
Angiogenesis genes				
*VEGFA*	rs3025039	0.14	0.139	0.159
*VEGFA*	rs2010963	0.497	0.33	0.15
*VEGFR2*	rs1870377	0.187	0.239	0.473

SNPs: Single nucleotide polymorphisms; DLBCL: diffuse large B-cell lymphoma.

### Correlations between SNPs and clinical/pathological characteristics of the DLBCL cohort

The study of correlations between SNPs and baseline clinical/pathological characteristics of patients showed associations between rs2010963 in the *VEGFA* gene and sex (*P* = 0.046, women showed a higher percentage of polymorphic genotypes compared with men) and between rs1625895 in the *TP53* gene and stage (*P* = 0.003, early stages showed a higher percentage of polymorphic genotypes compared with more advanced stages) [Table 2].

**Table 2:** Correlations between clinical/pathological characteristics of patients and study SNPs

**Characteristics**		**No. (%)**	**SNP**	***P* value**
**No.**		**185**		
Age, mean - SD		59.17-13.56	-	> 0.05
Gender	Male	93 (50.3)	rs2010963	0.046
	Female	92 (49.7)		
Stage	I	20 (10.8)	rs162589	0.003
	II	64 (34.6)		
	III	39 (21.1)		
	IV	62 (33.5)		
R-IPI	Very good (0)	26 (14.1)	-	> 0.05
	Good (1-2)	105 (56.8)		
	Poor (3-5)	54 (29.2)		
“B” symptoms	Present	41 (22.2)	-	> 0.05
	Absent	139 (75.1)		
	Missing	5 (2.7)		
Bulky disease	Yes	58 (31.4)	-	> 0.05
	No	122 (65.9)		
	Missing	5 (2.7)		
Bone marrow involvement	Yes	23 (12.4)	-	> 0.05
	No	140 (75.7)		
	Missing	22 (11.9)		
Performance status	0	115 (62.2)	-	> 0.05
	1	58 (31.4)		
	2	12 (6.5)		

SNPs: Single nucleotide polymorphisms; SD: standard deviation; R-IPI: revised International Prognostic Index.

### Correlations between SNPs and objective response of the DLBCL cohort

Overall, 176 out of 185 patients showed objective response [i.e., complete response (CR) *n* = 159 and partial response (PR) *n* = 17], as evaluated at the third and sixth cycles (end of treatment) of the R-CHOP regimen. Correlations between objective response and rs1800871 in the *IL10* gene were observed either according to an additive model [i.e., wild-type (GG) *vs.* heterozygous (GA) *vs.* mutant homozygote (AA) patients, *P* = 0.026] and a dominant model (GG + GA *vs.* AA patients, *P* = 0.020). According to rs1800871 genotypes, a higher response rate (96.2%) was observed in GG + GA patients compared with AA patients (81.2%). No statistically significant associations were found with the other 19 SNPs.

### Correlations between SNPs and survival parameters of the DLBCL cohort

Twenty SNPs previously identified as potential predictors of response to R-CHOP were evaluated. Thus, the associations of study SNPs with PFS and OS were assessed [Supplementary Tables 1-3]. After univariate analysis, a concordant effect represented by an increased risk of both disease progression and death was observed for two SNPs, i.e., rs1883112 in *NCF4* gene and rs1800871 in *IL10* gene [Table 3]. After multivariate analysis, both SNPs were successfully validated. A concordant effect of these two SNPs on PFS and OS was observed, according to an additive genetic model for rs1883112 in the *NCF4* gene and a dominant genetic model for rs1800871 in the *IL10* gene [Table 3].

**Table 3:** Significant associations between study SNPs and PFS or OS according to genetic models

**rs#**	**Gene**	Allelic change^*^	**PFS**				**OS**			
			**Model**	**HR**	**(95% CI)^a^**	***P***	**Model**	**HR**	**(95% CI)^a^**	***P***
**Univariate analysis^a^**										
rs1883112	*NCF4*	G > A	Additive	3.43	1.35, 8.71	0.009	Additive	3.94	1.14, 13.6	0.030
rs1800871	*IL10*	A > G	Dominant	1.75	1.18, 2.59	0.005	Dominant	2.05	1.32, 3.169	0.001
**Multivariate analysis^b^**										
rs1883112	*NCF4*	G > A	Additive	3.80	(1.50-9.65)	0.005	Additive	3.95	(1.14-13,7)	0.030
rs1800871	*IL10*	A > G	Dominant	4.01	(1.81-8.88)	< 0.001	Dominant	5.56	(2.28-13.6)	< 0.001

^a,b^Estimated through Cox proportional hazard model; ^*^https://www.ncbi.nlm.nih.gov/snp/. SNPs: Single nucleotide polymorphisms; PFS: progression-free survival; OS: overall survival; HR: hazard ratio; CI: confidence interval.

**Figure 1:**
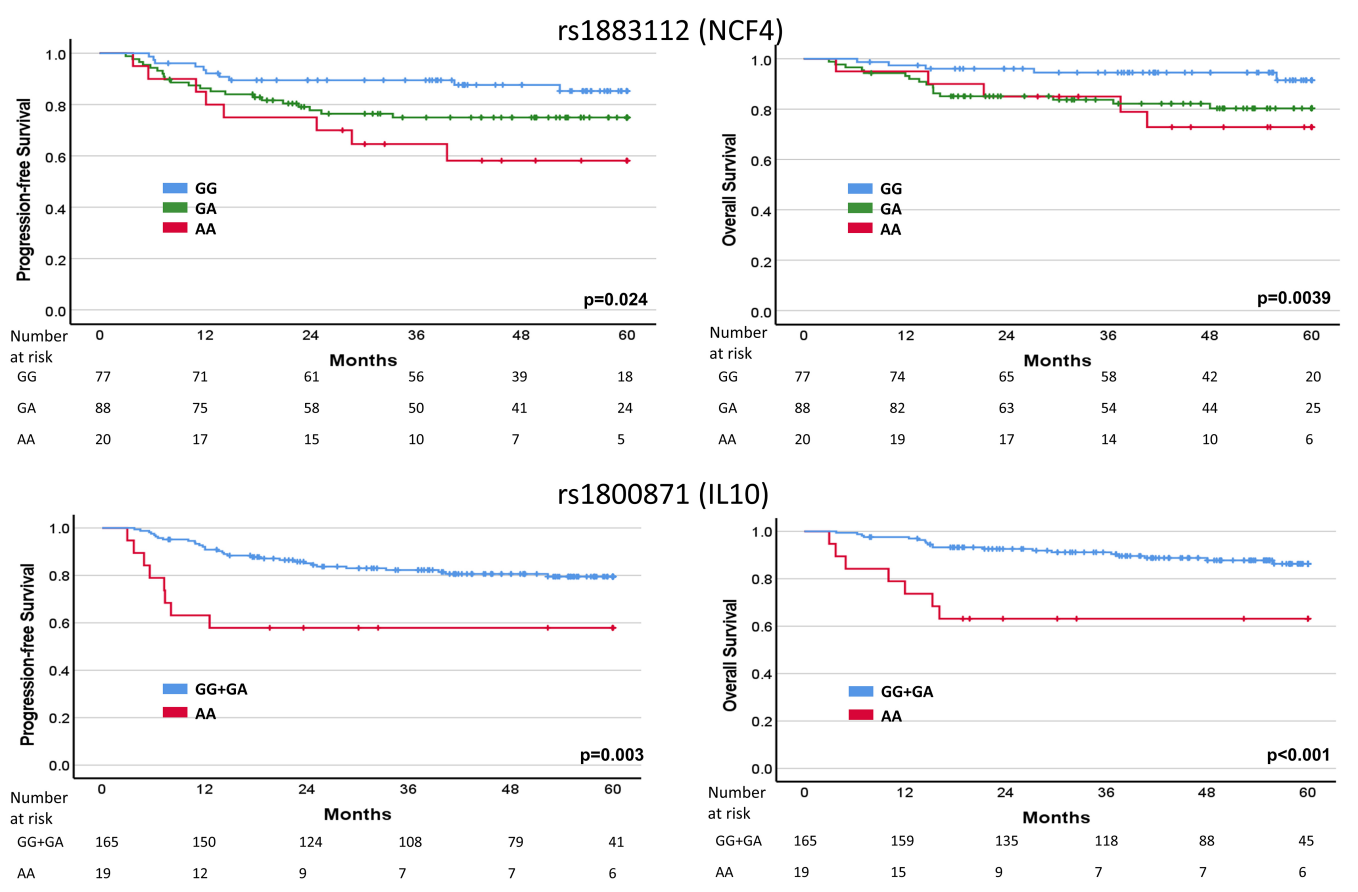
PFS and OS of the whole case series according to *NCF4* (additive model) and *IL10* (dominant model) SNPs (Kaplan-Meier method, log-rank Mantel-Cox test). PFS: Progression-free survival; OS: overall survival; SNPs: single nucleotide polymorphisms.

PFS and OS, according to *NCF4* and *IL10* SNPs, are reported in Figure 1. In relation to rs1883112 in the *NCF4* gene, patients harboring two rare alleles (AA) showed a shorter PFS and OS compared with wild-type (GG) or heterozygous (GA) patients. Concerning rs1800871 in the *IL10* gene, GG + GA patients showed a longer PFS and OS compared with AA patients.

### Correlations between *NCF4* rs1883112 or *IL10* rs1800871 SNPs and survival parameters according to R-IPI

**Figure 2:**
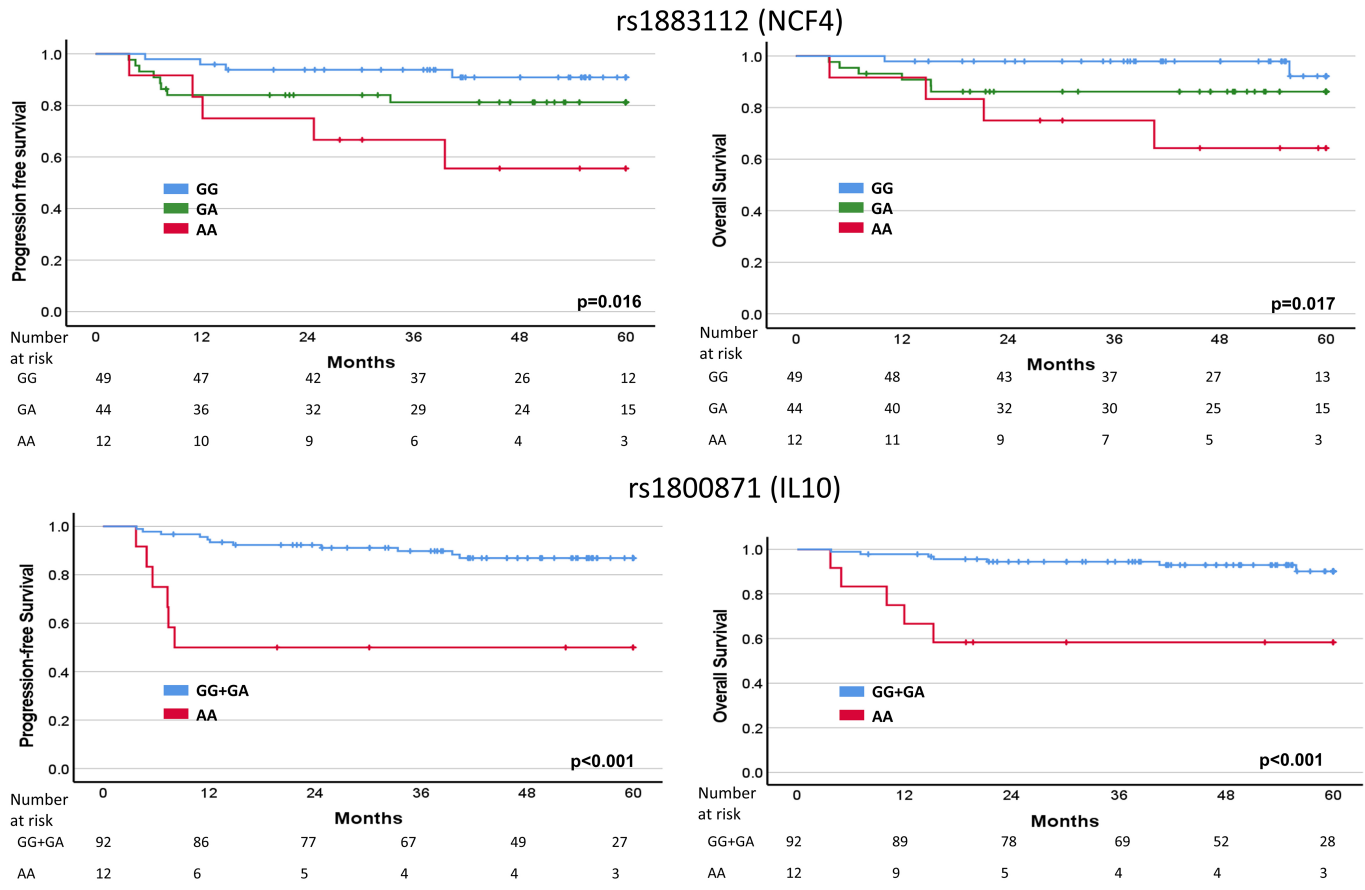
PFS and OS of patients with R-IPI 1-2 according to *NCF4* (additive model) and *IL10* (dominant model) SNPs (Kaplan-Meier method, log-rank Mantel-Cox test). PFS: Progression-free survival; OS: overall survival; R-IPI: revised International Prognostic Index; SNPs: single nucleotide polymorphisms.

Correlations between SNPs and PFS or OS were also analyzed by grouping patients according to R-IPI. Results showed that both SNPs were able to further discriminate PFS and OS of DLBCL patients belonging to the R-IPI 1-2 subgroup. In both cases, patients harboring two rare alleles (AA) showed a shorter PFS and OS compared with wild-type (GG) or heterozygous patients (GA) (i.e., *NCF4* rs1883112 according to the additive genetic model) or with wild-type (GG) plus heterozygous patients (GA) (i.e., *IL10* rs1800871 according to the dominant genetic model) [Figure 2]. No statistically significant difference was observed by grouping patients belonging to R-IPI 0 or R-IPI 3-5 subgroups.

## DISCUSSION

Tumor molecular characteristics and IPI greatly help to clinicians predict the patient’s prognosis. However, no patient’s germline characteristic is currently considered for this aim. Results of this validation study, obtained in a cohort of patients prospectively enrolled and homogeneously treated, show that host genetics may contribute to better predicting the efficacy of the R-CHOP regimen in DLBCL patients.

Through the years, a number of pharmacogenetic studies have been performed to establish the potential role of candidate SNPs in mediating resistance to R-CHOP, thus reducing its efficacy. SNPs that have been mainly investigated are present in genes that play a role in the pharmacokinetics/pharmacodynamics of drugs included in R-CHOP but also in the host immunity and in inflammatory processes. In this study, we planned to validate SNPs previously identified as biomarkers predictive of resistance to R-CHOP according to statistically significant associations. Among the 20 analyzed SNPs, two SNPs, i.e., rs1883112 in the *NCF4* gene and rs1800871 in the *IL10* gene, were associated with PFS and OS after univariate and multivariate analyses. rs1800871 in the *IL10* gene was also associated with the objective response to R-CHOP.

*NCF4* gene (i.e., neutrophil cytosol factor 4) is a cytosolic regulatory component of the superoxide-producing phagocyte NADPH-oxidase and thus plays a role in the host defense^[67]^. The rs1883112 SNP in the *NCF4* gene has been previously identified as a potential biomarker predictive of drug response in DLBCL patients by Liu *et al.*^[29]^. These authors showed that patients who carried the *NCF4* rs1883112 rare allele had significantly shorter PFS and event-free survival compared with those who carried the wild-type allele. This study was performed on 189 Asian patients whose clinical and pathological characteristics were highly comparable with those of our case series.

Interestingly, in a previous study including an identification (*n* = 337) and a validation cohort (*n* = 572) of patients with aggressive B-cell NHL treated with doxorubicin-based chemotherapy, only the rs1883112 SNP in the *NCF4* gene out of 53 candidate SNPs in 29 genes was successfully validated^[68]^. This *NCF4* SNP was found to be associated with PFS. As mentioned, all patients were treated with regimens including doxorubicin whose mechanism of action is dependent on the production of reactive oxygen species (ROS). Alterations in the promoter of the *NCF4* gene may be responsible for reduced production of ROS, thus for a decreased efficacy of doxorubicin that represents the backbone of the R-CHOP regimen^[55]^.

IL-10 is a cytokine with pleiotropic effects in immunoregulation and inflammation. Thus, SNPs in this gene may affect cytokine production by influencing the regulation of immune response. Therefore, the response to anticancer agents could be altered if combined with immunotherapeutic agents, including rituximab. It has been suggested that this monoclonal antibody can inhibit the IL10-mediated loops and downregulate BCL-2 expression, leading to tumor drug resistance reversal^[69]^. This mechanism could fail in the case of *IL10* genetic variants. An association between the rs1800871 *IL10* SNPs and PFS was found in a cohort of 337 DLBCL patients treated with R-CHOP^[64]^. Patients harboring the rare allele showed a shorter PFS.

In this study, we have also shown that the rs1883112 SNP in the *NCF4* gene and rs1800871 in the *IL10* were able to further discriminate PFS and OS of DLBCL patients belonging to the R-IPI 1-2 subgroup. In both cases, homozygous mutant patients showed a shorter PFS and OS compared with wild-type or heterozygous patients. Thus, although R-IPI 1-2 categorizes patients as having a good prognosis, our results show that patients with two rare alleles are more likely to develop tumor drug resistance, as suggested by their worst prognosis compared with patients with the other genotypes. Thus, the R-IPI stratification, currently based on hematochemical, clinical, and pathological features, could benefit from the addition of genetic characteristics that are able to further improve the prediction of drug response and prognosis.

We also found statistically significant associations between rs2010963 in the *VEGFA* gene and sex and between rs1625895 in the *TP53* gene and stage.

Some angiogenesis genetic variants have been proposed to affect the clinical features and the prognosis of DLBCL patients treated with R-CHOP^[30]^. We evaluated the potential predictive role of three SNPs involved in angiogenesis, i.e., *VEGFA* rs3025039, *VEGFA* rs2010963, and *VEGFR2* rs870377, and we found a correlation between *VEGFA* rs2010963 and sex. To date, the effects of this SNP variant on the VEGFA protein have not been fully clarified^[70]^, and previous data on this association are not available.

Through the years, the role of TP53 in protecting against tumor growth and in promoting cellular DNA repair, apoptosis, and other fundamental processes useful to counteract tumor cells has been widely recognized, and this tumor suppressor gene is today known as the guardian of the human genome^[71]^. Additionally, the overexpression of mutated *TP53*, leading to the reduction or abolition of its function, has been frequently related to resistance to anticancer drugs, including cytotoxic and targeted agents^[72]^. *TP53* SNPs have been previously suggested as predictive biomarkers in DLBCL patients treated with R-CHOP^[28]^. *TP53* rs1625895 is an intronic polymorphism with potential effects on the *TP53* expression levels and function by interfering with RNA splicing and by the interaction between DNA strands and proteins. We found that the rare allele was mainly harbored by patients at the early stages of disease. To our knowledge, this is the first time that this SNP has been found to be associated with stage in DLBCL patients. However, this finding is in agreement with that of Kochethu *et al.*^[73]^, who reported that the rare allele was associated with the initial stages of chronic lymphocytic leukemia. Interestingly, these authors, in agreement with our results, found no association between *TP53* rs1625895 SNP and survival parameters. Current knowledge is insufficient to suggest potential explanations for the identified correlation between *TP53* rs1625895 SNP and stage.

### Limitations

Concerning the potential limitations of this study, the following considerations may be provided. This study was carried out in a Caucasian population. The homogeneity of this case series may represent an advantage in terms of the reliability of results due to the lack of the potential impact related to the MAF variability among populations of different ethnicities. However, this advantage may also become a limit since, in the meantime, such homogeneity prevents the capture of potential differences among populations. Thus, to further understand the role of the study SNPs in R-CHOP response in DLBCL patients, it would be useful to validate them also in populations other than Caucasians.

A second aspect is that study SNPs had been evaluated in the original studies by different methodologies (i.e., TaqMan allelic discrimination assays, Sanger sequencing, targeted sequencing, *etc.*). Thus, an option could have been represented by the validation of such SNPs with the respective methods used for the identification phase. However, the use of a high throughput sensitive and accurate approach such as that we used facilitates the standardization of results, thus avoiding potential variabilities related to the different identification strategies, and offers valuable insights into the generalizability of these findings even across different populations (when available) and study designs.

### Conclusions

Overall, we were able to investigate for validation more than 70% of all the SNPs that, through the years, have been suggested as potential predictors of R-CHOP efficacy in DLBCL patients and we validated two of them. This validation study is based on a DLBCL cohort of patients who have been prospectively enrolled and homogeneously treated with R-CHOP. This cohort appears to be a good representative of a DLBCL population in terms of survival^[57]^. Additionally, the MAF of the study SNPs in this cohort was highly comparable to that of Caucasian subjects.

Thus, the results of this study support the already suggested predictive role of response to R-CHOP of rs1883112 in the *NCF4* gene and rs1800871 in the *IL10* gene and highlight their ability to further discriminate the prognosis of patients belonging to the R-IPI 1-2 subgroup. These data point to the necessity of also focusing on host genetics, possibly taking into account genotypes of rs1883112 in the *NCF4* gene and rs1800871 in the *IL10* gene, for a more comprehensive assessment of the outcomes of DLBCL patients in future prospective clinical pharmacogenetic trials.

## DECLARATIONS

### Availability of data and materials

Genotyping data were from the array available on the NCBI GEO repository as GSE186441^[57]^.

### Copyright

© The Author(s) 2024.
