# Supplementary material for: Validation of single nucleotide polymorphisms potentially related to R-CHOP resistance in diffuse large B-cell lymphoma patients
Source: Cancer Drug Resist. 2024 May 24;7:21. doi: 10.20517/cdr.2024.10 (PMC11149109; doi:10.20517/cdr.2024.10)
Supplement: Supplementary file 1 [file cdr-7-21-SupplementaryMaterials.pdf]

Supplementary Materials

Validation of single nucleotide polymorphisms potentially related to R-CHOP resistance in diffuse large B-cell lymphoma patients

Gabriele Perrone[1], Luigi Rigacci[2], Giandomenico Roviello[1], Ida Landini[1], Alberto Fabbri[3], Lorenzo Iovino[4,5], Benedetta Puccini[6], Emanuele Cencini[3], Enrico Orciuolo[4], Monica Bocchia[3], Alberto Bosi[7], Enrico Mini[1,#], Stefania Nobili[8,#]

[1]Department of Health Sciences, University of Florence, Florence 50139, Italy.
[2]Research Unit of Hematology, Department of Medicine and Surgery, Campus Biomedico University, Rome 00128, Italy.
[3]Unit of Hematology, Azienda Ospedaliera Universitaria Senese, University of Siena, Siena 53100, Italy.
[4]Unit of Hematology, Santa Chiara University Hospital, University of Pisa, Pisa 56126, Italy.
[5]Clinical Research Division, Fred Hutchinson Cancer Center, Seattle, WA 98109-4433, USA.
[6]Unit of Hematology, Careggi University-Hospital, Florence 50134, Italy.
[7]Department of Experimental and Clinical Medicine, University of Florence, Florence 50134, Italy.
[8]Department of Neuroscience, Psychology, Drug Research and Child Health, University of Florence, Florence 50139, Italy.
[#]Authors contributed equally.

Correspondence to: Dr. Stefania Nobili, Department of Neuroscience, Psychology, Drug Research and Child Health - NEUROFARBA, University of Florence, Viale Pieraccini, 6, Florence 50139, Italy. E-mail: stefania.nobili@unifi.it; Prof. Enrico Mini, Department of Health Sciences, University of Florence, Viale Pieraccini, 6, Firenze 50139, Italy. E-mail: enrico.mini@unifi.it

Supplementary Table 1. Associations between genetic polymorphisms and progression free survival or overall survival (univariate analysis)

Additive model						
	PFS			OS		
SNP	HR[1]	95% CI[1]	p-value	HR[1]	95% CI[1]	*P*-value
rs17222723						
Wt	—	—		—	—	
Het	0.80	0.33, 1.91	0.6	0.58	0.17, 1.93	0.4
Mut	0.96	0.13, 7.01	>0.9	1.50	0.20, 11.1	0.7
rs1695						
Wt	—	—		—	—	
Het	1.19	0.63, 2.28	0.6	1.23	0.56, 2.71	0.6
Mut	0.45	0.10, 1.92	0.3	0.34	0.04, 2.61	0.3
rs1883112						
Wt	—	—		—	—	
Het	1.99	0.94, 4.23	0.073	3.03	1.11, 8.26	0.031
Mut	3.43	1.35, 8.71	0.009	3.94	1.14, 13.6	0.030
rs4673						
Wt	—	—		—	—	
Het	0.80	0.41, 1.57	0.5	0.69	0.31, 1.53	0.4
Mut	0.97	0.35, 2.63	>0.9	0.49	0.11, 2.20	0.4
rs1041981						
Wt	—	—		—	—	
Het	0.81	0.42, 1.58	0.5	1.25	0.57,	0.6

					2.75	
Mut	1.13	0.38, 3.32	0.8	0.50	0.06, 3.90	0.5
rs1625895						
Wt	—	—		—	—	
Het	1.19	0.60, 2.36	0.6	1.50	0.66, 3.44	0.3
Mut	1.05	0.25, 4.43	>0.9	1.72	0.39, 7.55	0.5
rs1800871						
Wt	—	—		—	—	
Het	0.84	0.41, 1.70	0.6	0.77	0.31, 1.91	0.6
Mut	2.80	1.21, 6.50	0.016	3.74	1.45, 9.66	0.007
rs1870377						
Wt	—	—		—	—	
Het	1.18	0.61, 2.32	0.6	1.57	0.72, 3.42	0.3
Mut	0.83	0.11, 6.12	0.9	0.00	0.00, Inf	>0.9
rs2231142						
Wt	—	—		—	—	
Het	1.12	0.40, 3.16	0.8	0.78	0.18, 3.33	0.7
Mut	46.0	5.13, 413	<0.001	59.6	6.18, 575	<0.001
rs20572						
Wt	—	—		—	—	
Het	1.25	0.52, 2.99	0.6	1.63	0.61, 4.33	0.3
rs9024						
Wt	—	—		—	—	
Het	1.25	0.52, 2.99	0.6	1.63	0.61,	0.3

					4.33	
rs13058338						
Wt	—	—		—	—	
Het	1.72	0.90, 3.32	0.10	1.56	0.69, 3.53	0.3
Mut	1.38	0.41, 4.68	0.6	2.04	0.58, 7.24	0.3
rs1385129						
Wt	—	—		—	—	
Het	0.51	0.23, 1.16	0.11	0.31	0.09, 1.04	0.057
Mut	0.58	0.08, 4.22	0.6	0.84	0.11, 6.27	0.9
rs841853						
Wt	—	—		—	—	
Het	1.05	0.51, 2.16	0.9	1.25	0.50, 3.14	0.6
Mut	1.87	0.76, 4.58	0.2	2.38	0.80, 7.09	0.12
rs11549467						
Wt	—	—		—	—	
Het	183	11.5, 2,934	<0.001	183	11.5, 2,934	<0.001
rs1130409						
Wt	—	—		—	—	
Het	1.00	0.49, 2.02	>0.9	1.37	0.55, 3.40	0.5
Mut	0.89	0.35, 2.22	0.8	1.18	0.37, 3.72	0.8
rs3025039						
Wt	—	—		—	—	
Het	1.06	0.52, 2.18	0.9	1.34	0.58,	0.5

					3.08	
Mut	0.00	0.00, Inf	>0.9	0.00	0.00, Inf	>0.9
rs2010963						
Wt	—	—		—	—	
Het	0.82	0.42, 1.61	0.6	0.55	0.24, 1.27	0.2
Mut	0.65	0.24, 1.79	0.4	0.67	0.22, 2.08	0.5
rs10276036						
Het	—	—		—		
Mut	1.05	0.51, 2.14	>0.9	0.67	0.26, 1.72	0.4
Wt	0.49	0.21, 1.17	0.11	0.48	0.18, 1.33	0.2
rs1128503						
Het	—	—		—	—	
Mut	1.22	0.59, 2.53	0.6	0.75	0.29, 1.95	0.6
Wt	0.53	0.22, 1.28	0.2	0.50	0.18, 1.39	0.2

1 HR = Hazard Ratio, CI = Confidence Interval

Supplementary Table 2. Associations between genetic polymorphisms and progression free survival or overall survival (univariate analysis)

Recessive model						
	PFS			OS		
**SNP**	**HR[1]**	**95% CI[1]**	***P*-value**	**HR[1]**	**95% CI[1]**	***P*-value**
rs17222723						
Wt	—	—		—	—	
Het+Mut	0.82	0.36, 1.86	0.6	0.68	0.24, 1.98	0.5
rs1695						
Wt	—	—		—	—	
Het+Mut	1.03	0.55, 1.93	>0.9	1.04	0.48, 2.25	>0.9
rs1883112						
Wt	—	—		—	—	
Het+Mut	1.50	1.05, 2.15	0.027	1.789	1.09, 2.94	0.19
rs4673						
Wt	—	—		—	—	
Het+Mut	0.83	0.44, 1.57	0.6	0.65	0.30, 1.40	0.3
rs1041981						
Wt	—	—		—	—	
Het+Mut	0.86	0.46, 1.61	0.6	1.14	0.52, 2.47	0.7
rs1625895						
Wt	—	—		—	—	
Het+Mut	1.17	0.61, 2.24	0.6	1.54	0.71, 3.36	0.3
rs1800871						
Wt	—	—		—	—	
Het+Mut	1.06	0.77, 1,45	0.721	1.10	0.75, 1.63	0.61
rs1870377						
Wt	—	—		—	—	
Het+Mut	1.15	0.60, 2.21	0.7	1.42	0.65, 3.09	0.4
rs2231142						
Wt	—	—		—	—	
Het+Mut	1.39	0.54, 3.56	0.5	1.17	0.35, 3.90	0.8

rs20572						
	—	—		—	—	
	—	—		—	—	
rs9024						
	—	—		—	—	
	—	—		—	—	
rs13058338						
Wt	—	—		—	—	
Het+Mut	1.66	0.89, 3.12	0.11	1.64	0.76, 3.54	0.2
rs1385129						
Wt	—	—		—	—	
Het+Mut	0.52	0.24, 1.13	0.10	0.37	0.13, 1.07	0.1
rs841853						
Wt	—	—		—	—	
Het+Mut	1.21	0.61, 2.38	0.6	1.47	0.62, 3.50	0.4
rs11549467						
	—	—		—	—	
	—	—		—	—	
rs1130409						
Wt	—	—		—		
Het+Mut	0.96	0.49, 1.87	>0.9	1.31	0.55, 3.13	0.5
rs3025039						
Wt	—	—		—		
Het+Mut	1.0	0.48, 2.04	>0.9	1.26	0.55, 2.91	0.6
rs2010963						
Wt	—	—		—		
Het+Mut	0.78	0.41, 1.48	0.4	0.58	0.27, 1.25	0.2
rs10276036						
Wt	—	—		—		
Het+Mut	2.06	0.91, 4.66	0.084	1.81	0.68, 4.81	0.2
rs1128503						

Wt	—	—		—	—	
Het+Mut	2.02	0.89, 4.60	0.094	1.82	0.68, 4.85	0.2

1 HR = Hazard Ratio, CI = Confidence Interval

Supplementary Table 3. Associations between genetic polymorphisms and progression free survival or overall survival (univariate analysis)

Dominant model						
	PFS			OS		
SNP	HR[1]	95% CI[1]	*P*-value	HR[1]	95% CI[1]	*P*-value
rs17222723						
Wt+Het	—	—		—	—	
Mut	1.00	0.14, 7.25	>0.9	1.63	0.22, 12.1	0.6
rs1695						
Wt+Het	—	—		—	—	
Mut	0.37	0.09, 1.60	0.2	0.28	0.04, 2.12	0.2
rs1883112						
Wt+Het	—	—		—	—	
Mut	1.51	1.02, 2.24	0.039	1.39	0.85, 2.26	0.186
rs4673						
Wt+Het	—	—		—	—	
Mut	1.09	0.43, 2.80	0.9	0.61	0.14, 2.57	0.5
rs1041981						
Wt+Het	—	—		—	—	
Mut	1.25	0.44, 3.52	0.7	0.45	0.06, 3.30	0.4
rs1625895						
Wt+Het	—	—		—	—	
Mut	1.00	0.24, 4.13	>0.9	1.51	0.36, 6.38	0.6
rs1800871						
Wt+Het	—	—		—	—	
Mut	1.75	1.18,	0.005	2.05	1.32,	0.001

		2.59			3.169	
rs1870377						
Wt+Het	—	—		—	—	
Mut	0.78	0.11, 5.72	0.8	0.00	0.00, Inf	>0.9
rs2231142						
Wt+Het	—	—		—	—	
Mut	45.5	5.09, 407	<0.001	60.8	6.33, 585	<0.001
rs20572						
	—	—		—	—	
	—	—		—	—	
rs9024						
	—	—		—	—	
	—	—		—	—	
rs13058338						
Wt+Het	—	—		—	—	
Mut	1.09	0.34, 3.53	0.9	1.69	0.51, 5.64	0.4
rs1385129						
Wt+Het	—	—		—	—	
Mut	0.68	0.09, 4.94	0.7	1.07	0.14, 7.89	>0.9
rs841853						
Wt+Het	—	—		—	—	
Mut	1.82	0.84, 3.96	0.13	2.07	0.83, 5.16	0.12
rs11549467						
	—	—		—	—	
	—	—		—	—	
rs1130409						
Wt+Het	—	—		—	—	

Mut	0.89	0.39, 2.01	0.8	0.97	0.36, 2.56	>0.9
rs3025039						
Wt+Het	—	—		—	—	
Mut	0.00	0.00, Inf	>0.9	0.00	0.00, Inf	>0.9
rs2010963						
Wt+Het	—	—		—	—	
Mut	0.73	0.29, 1.87	0.5	0.92	0.32, 2.67	0.9
rs10276036						
Wt+Het	—	—		—	—	
Mut	1.32	0.67, 2.61	0.4	0.85	0.34, 2.11	0.7
rs1128503						
Wt+Het	—	—		—	—	
Mut	1.52	0.76, 3.02	0.2	0.95	0.38, 2.37	>0.9

1 HR = Hazard Ratio, CI = Confidence Interval
